# Solriamfetol for Excessive Daytime Sleepiness in Parkinson's Disease: Phase 2 Proof‐of‐Concept Trial

**DOI:** 10.1002/mds.28702

**Published:** 2021-06-30

**Authors:** Aleksandar Videnovic, Amy W. Amara, Cynthia Comella, Paula K. Schweitzer, Helene Emsellem, Kris Liu, Amanda L. Sterkel, Mildred D. Gottwald, Joshua R. Steinerman, Philip Jochelson, Katie Zomorodi, Robert A. Hauser

**Affiliations:** ^1^ Movement Disorders Unit and Division of Sleep Medicine Massachusetts General Hospital Boston Massachusetts USA; ^2^ Division of Movement Disorders, Department of Neurology University of Alabama at Birmingham Birmingham Alabama USA; ^3^ Parkinson's Disease and Movement Disorders Program Rush University Chicago Illinois USA; ^4^ Sleep Medicine and Research Center St. Luke's Hospital Chesterfield Missouri USA; ^5^ The Center for Sleep & Wake Disorders Chevy Chase Maryland USA; ^6^ Jazz Pharmaceuticals Palo Alto California USA; ^7^ Jazz Pharmaceuticals Philadelphia Pennsylvania USA; ^8^ Parkinson's Disease and Movement Disorders Center University of South Florida Tampa Florida USA

**Keywords:** JZP‐110, Sunosi, Parkinson's disease, sleep‐wake disorders, therapeutics

## Abstract

**Background:**

Solriamfetol is approved (US and EU) for excessive daytime sleepiness (EDS) in narcolepsy and obstructive sleep apnea.

**Objectives:**

Evaluate solriamfetol safety/efficacy for EDS in Parkinson's disease (PD).

**Methods:**

Phase 2, double‐blind, 4‐week, crossover trial: adults with PD and EDS were randomized to sequence A (placebo, solriamfetol 75, 150, 300 mg/d), B (solriamfetol 75, 150, 300 mg/d, placebo), or C (placebo). Outcomes (safety/tolerability [primary]; Epworth Sleepiness Scale [ESS]; Maintenance of Wakefulness Test [MWT]) were assessed weekly. *P* values are nominal.

**Results:**

Common adverse events (n = 66): nausea (10.7%), dizziness (7.1%), dry mouth (7.1%), headache (7.1%), anxiety (5.4%), constipation (5.4%), dyspepsia (5.4%). ESS decreased both placebo (−4.78) and solriamfetol (−4.82 to −5.72; *P >* 0.05). MWT improved dose‐dependently with solriamfetol, increasing by 5.05 minutes with 300 mg relative to placebo (*P =* 0.0098).

**Conclusions:**

Safety/tolerability was consistent with solriamfetol's known profile. There were no significant improvements on ESS; MWT results suggest possible benefit with solriamfetol in PD. © 2021 The Authors. *Movement Disorders* published by Wiley Periodicals LLC on behalf of International Parkinson and Movement Disorder Society

Excessive daytime sleepiness (EDS), a nonmotor symptom affecting 20%–60% of patients with Parkinson's disease (PD),^1^ may be a manifestation of the primary pathology of PD^2^, ^3^, ^4^ and/or secondary to soporific effects of dopaminergic medication, coexisting sleep disorders, psychiatric comorbidities, autonomic dysfunction, and other unknown factors.^3^, ^4^, ^5^

Regardless of etiology, the disabling consequences of EDS in PD include undesired sleep episodes, reduced attention, cognitive impairment, effects on mood, increased accidents, decreased productivity, and impaired functioning.^1^, ^6^, ^7^, ^8^

There is currently no approved pharmacologic treatment for EDS in PD in the United States (US) or European Union (EU). Treatment guidelines for managing EDS in PD suggest that modafinil may be “possibly useful,”^9^, ^10^ with evidence suggesting that modafinil may improve patients' perception of sleepiness, but not objective sleepiness.^9^, ^10^, ^11^, ^12^, ^13^ Stimulants may have modest benefits, but adverse effects.^3^

Solriamfetol, a dopamine/norepinephrine reuptake inhibitor,^14^ is approved in the US and EU (Sunosi™) to treat EDS in adults with narcolepsy (75–150 mg/d) or obstructive sleep apnea (OSA; 37.5–150 mg/d).^15^, ^16^ Based on the unmet need for pharmacologic treatment for EDS in PD patients, this study sought to characterize the safety and efficacy of solriamfetol in this population.

## Methods

### Study Design

This phase 2, randomized, double‐blind, placebo‐controlled, crossover trial was conducted at 33 US sites between February 2017 and August 2018.

Participants (aged 35–80 years) with PD (United Kingdom Parkinson's Disease Society Brain Bank criteria) and EDS (Epworth Sleepiness Scale [ESS] score > 11) were randomized 3:3:1 to one of three treatment sequences (A, B, C) (Fig. S1A). Each sequence included four 1‐week periods, during which participants received once‐daily oral solriamfetol or placebo; there was no washout between periods. In sequences A and B, solriamfetol was administered in an upward titration (A: placebo, 75, 150, 300 mg; B: 75, 150, 300 mg, placebo). Participants in sequence C received placebo during all four periods.

### Outcome Measures

Safety, efficacy, and pharmacokinetics (PK) were assessed at the end of each 1‐week treatment period. Primary outcomes were safety/tolerability based on adverse events (AEs), clinical laboratory findings, vital signs, electrocardiograms (ECGs), and the Columbia‐Suicide Severity Rating Scale (C‐SSRS). Secondary outcomes included change from baseline in ESS score and PK. Exploratory outcomes included changes from baseline in Maintenance of Wakefulness Test (MWT)^17^ mean sleep latency, percentage of participants improved on the Patient and Clinician Global Impression of Change (PGI‐C and CGI‐C, respectively), PD motor symptoms (Movement Disorder Society–Unified Parkinson's Disease Rating Scale [MDS‐UPDRS] Parts III and IV), and nonmotor symptoms (Fatigue Severity Scale [FSS], Apathy Scale, and Scales for Outcomes in Parkinson's Disease‐Cognition [SCOPA‐COG]).

### Statistical Analysis

Statistical methods are described in Appendix S1.

## Results

### Participant Population

The safety population included 66 participants; 64 comprised the modified intent‐to‐treat population and 62 (93.9%) completed the study (Fig. S1B **)**. Demographic and baseline clinical characteristics were generally similar across sequence groups (Table S1). All participants were receiving levodopa and/or dopamine agonists at baseline; 28 were taking dopamine agonists; 20 were on monoamine oxidase‐B (MAO‐B) inhibitors (Table S1).

### Safety and Tolerability

Thirty‐three (58.9%) participants had ≥1 treatment‐emergent AE (TEAE) while taking solriamfetol; the most frequent were nausea, dizziness, dry mouth, headache, anxiety, constipation, and dyspepsia (Table 1). Most TEAEs were mild or moderate in severity; the only severe TEAE (hypertension) was deemed unrelated to treatment. One participant had two serious TEAEs (hematuria, asthenia/generalized weakness), which occurred during the safety follow‐up period after treatment with solriamfetol 300 mg and were considered, by the investigator, unrelated to treatment. Three participants discontinued because of AEs, which occurred during solriamfetol treatment (freezing phenomenon [75 mg], anxiety [150 mg], and balance disorder [150 mg]). There were no life‐threatening or fatal AEs and no findings of suicidal ideation or behavior on the C‐SSRS. TEAEs by dopamine agonist use are summarized in Table S2. There were minor or no clinically meaningful changes in blood pressure (Table S3 **)**, clinical laboratory findings, or ECG qualitative parameters.

**TABLE 1:** Treatment‐emergent adverse events (safety population)

Participants with ≥1 TEAE, n (%)	Placebo	Solriamfetol			
		75 mg	150 mg	300 mg	Combined^a^
	(n = 64)	(n = 56)	(n = 55)	(n = 54)	(n = 56)
Any TEAE	16 (25.0)	18 (32.1)	20 (36.4)	15 (27.8)	33 (58.9)
Mild or moderate TEAE	15 (23.4)	18 (32.1)	20 (36.4)	15 (27.8)	33 (58.9)
TEAE related to study drug	8 (12.5)	10 (17.9)	10 (18.2)	7 (13.0)	19 (33.9)
Serious TEAE	0	0	0	1 (1.9)	1 (1.8)
Discontinuation due to TEAE^b^	0	1 (1.8)	2 (3.6)	0	3 (5.4)
Common TEAEs (≥5%)					
Nausea	0	2 (3.6)	2 (3.6)	3 (5.6)	6 (10.7)
Dizziness	0	3 (5.4)	0	1 (1.9)	4 (7.1)
Dry mouth	2 (3.1)	2 (3.6)	2 (3.6)	0	4 (7.1)
Headache	0	1 (1.8)	3 (5.5)	2 (3.7)	4 (7.1)
Anxiety	2 (3.1)	1 (1.8)	2 (3.6)	0	3 (5.4)
Constipation	0	2 (3.6)	1 (1.8)	0	3 (5.4)
Dyspepsia	0	2 (3.6)	1 (1.8)	0	3 (5.4)

^a^ Pooled across all solriamfetol doses.

^b^ TEAE leading to early discontinuation of study drug and/or to study withdrawal.

Abbreviation: TEAE, treatment‐emergent adverse event.

### Efficacy

**FIG. 1:**
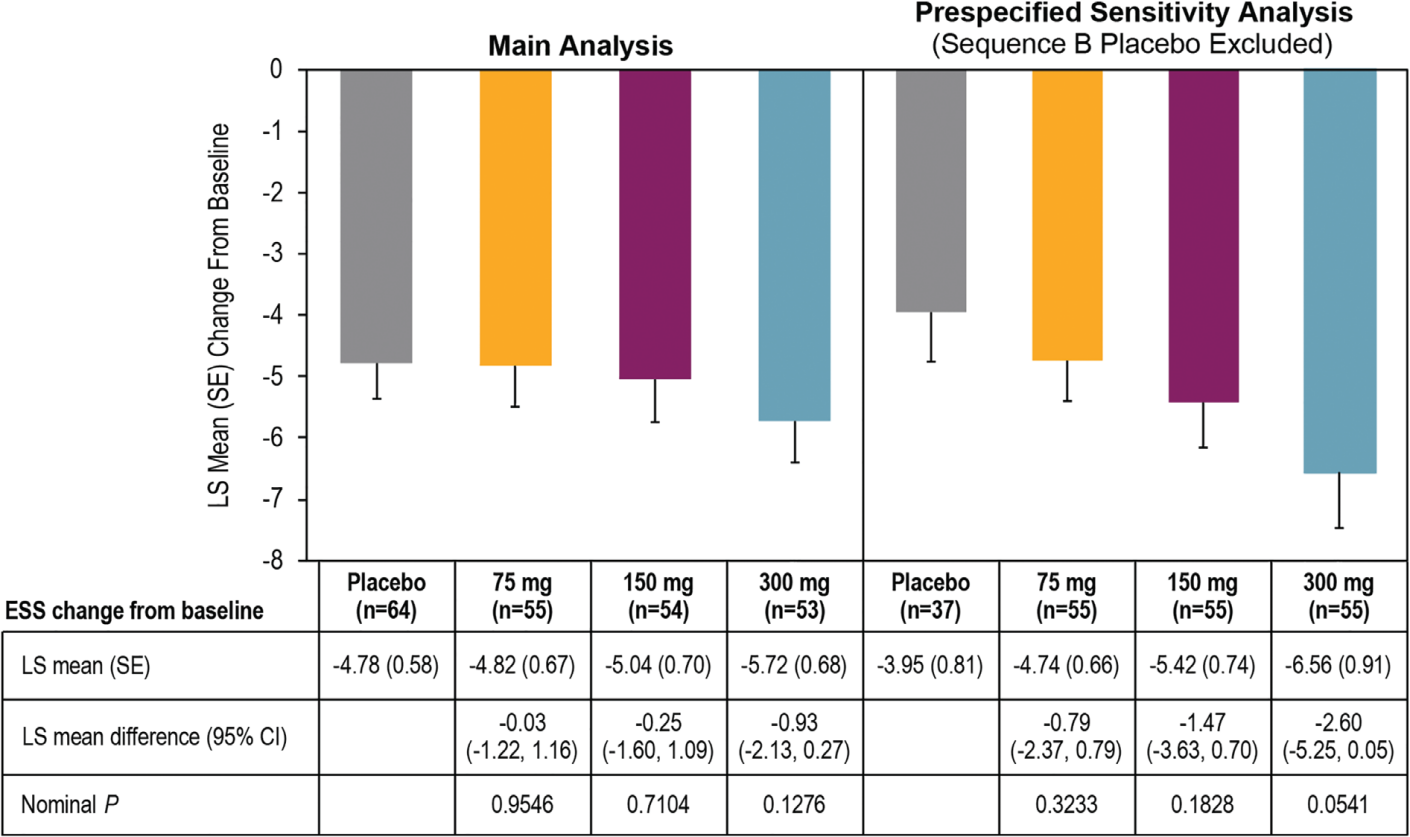
Change from baseline on the secondary efficacy endpoint, Epworth Sleepiness Scale scores (modified intent‐to‐treat population). Note :  Placebo group represents a single pooled placebo group (combination of placebo groups from each treatment sequence). To account for potential carryover effects on ESS from the 300 mg to placebo period in sequence B, given the 1‐week recall period and the lack of washout between treatment periods, a prespecified sensitivity analysis for the ESS excluded sequence B placebo data. LS, least squares; SE, standard error; ESS, Epworth Sleepiness Scale; CI, confidence interval.

For the ESS, least squares (LS) mean changes from baseline ranged from −4.82 to −5.72 across solriamfetol doses compared with −4.78 with placebo (Fig. 1). It was suspected that the placebo group may have been impacted by carryover effects in sequence B (when the placebo period followed the 300 mg period). Indeed, mean change from baseline was greater when the placebo period followed the 300 mg period (sequence B; −6.5) than when the placebo period occurred in week 1 (sequence A; −3.6). In the prespecified sensitivity analysis that excluded sequence B placebo, the LS mean change from baseline with placebo was smaller (−3.95), with greater, dose‐dependent differences between placebo and solriamfetol relative to the main analysis (Fig. 1). Greater treatment effects were observed in participants receiving dopamine agonists (Fig. S2).

MWT mean sleep latency showed dose‐dependent improvements with solriamfetol, increasing by 5.05 minutes with 300 mg relative to placebo (95% confidence interval [CI], 1.24 to 8.57; *P* = 0.0098) (Table S4). Greater treatment effects were observed in participants not receiving dopamine agonists (Table S4).

Across solriamfetol doses, 52.7%–65.5% of participants were improved on PGI‐C and 56.4%–67.3% on CGI‐C; 60.9% improved with placebo on both scales (Table S4). Post hoc analyses of PGI‐C are shown in Table S4.

There was no worsening in PD motor symptoms (MDS‐UPDRS Parts III/IV) or nonmotor symptoms (Apathy Scale, FSS, and SCOPA‐COG) with solriamfetol relative to placebo (Table S4).

Per protocol analyses of ESS and MWT are shown in Table S5.

### Pharmacokinetics

Solriamfetol was rapidly absorbed (median time to reach maximum plasma concentration [T_max_] 1.1 to 2.0 hours) with dose‐proportional exposure increases at steady state and similar mean elimination half‐life across doses (8.0 to 9.5 hours; Fig. S3; Table S6).

## Discussion

This was the first study to assess solriamfetol treatment in participants with EDS and PD. No new AEs or safety concerns were identified in this population compared with the known safety profile of solriamfetol in narcolepsy and OSA.^18^, ^19^ Additionally, this is the first investigation of solriamfetol in participants using concomitant dopaminergic agents. All participants were on dopaminergic therapy (mean levodopa equivalent dose 623 mg). Coadministration of both agents in this short‐term study did not lead to an AE profile different from solriamfetol monotherapy in narcolepsy or OSA. Importantly, solriamfetol is contraindicated with MAO inhibitors, and 20 participants were on MAO‐B inhibitors in this study.^15^ Long‐term trials in larger samples are needed to further evaluate the safety and tolerability of solriamfetol with concomitant dopaminergic agents or MAO inhibitors.

There were no statistical differences in ESS scores with solriamfetol compared with placebo; however, additional analyses revealed that the main analysis was likely confounded by carryover effects. Similar carryover effects were observed on PGI‐C. The placebo response observed on self‐report measures (ESS and PGI‐C) was not evident on MWT. The main analysis for MWT showed dose‐dependent effects, and solriamfetol 300 mg increased sleep latency by 5 minutes compared with placebo. A minimum clinically important change in MWT sleep latency has not been established in patients with PD. However, in participants with narcolepsy or OSA, an increase of 4 minutes is associated with PGI‐C ratings of “minimally improved” or better, and an increase of 7 minutes is associated with ratings of “much improved” or better.^20^ Solriamfetol's PK profile was consistent with previous reports in healthy volunteers and participants with narcolepsy or OSA, suggesting differences in efficacy are likely not attributable to differences in solriamfetol exposure.^21^

Improvement on ESS was greater among those taking dopamine agonists, while improvement on MWT was greater among those not taking these agents. Although use of dopamine agonists is associated with subjective sleepiness, it is difficult to speculate about the reasons for these apparent differences. These findings illustrate the challenge of using outcome measures that only weakly to moderately correlate.^22^, ^23^

Other agents have been evaluated in PD patients with EDS in studies with varying designs and methodologies that, generally, included small sample sizes; larger trials are warranted. Modafinil improved ESS scores in some,^12^, ^24^ but not all,^11^ studies; a meta‐analysis found an overall treatment effect of −2.24 (95% CI −3.90 to −0.57) for modafinil versus placebo.^13^ Modafinil has not demonstrated improvements on MWT^12^ or mean sleep latency test [MSLT].^11^ Sodium oxybate significantly improved ESS scores (−4.2 vs. placebo) and MSLT sleep latency (+2.9 minutes vs. placebo), but has been associated with small, though significant, increases in apneic events.^25^, ^26^ Atomoxetine significantly improved ESS scores (−2.9 vs. placebo) in patients with PD and depression.^27^ Finally, caffeine showed minimal improvement on ESS (mean difference −1.71); findings were not significant.^28^

A limitation of this study is the lack of washout periods between treatments. One previous phase 2a narcolepsy study demonstrated efficacy of solriamfetol using a crossover design with 2‐week treatment periods (and did not suggest carryover effects).^29^ Further, solriamfetol is rapidly absorbed (median T_max_ ~2 hours), with a mean elimination half‐life of ~6 hours.^30^ Considering those data, washout periods were not included in this study. However, as noted, sensitivity analyses suggested a carryover effect in the group that received placebo immediately after solriamfetol (300 mg/d). The 1‐week recall for the ESS likely exacerbated carryover effects.

Additional limitations include the small sample size, which had insufficient power to detect statistical differences between groups in the dopamine agonist use subgroup analyses. Larger studies are needed to further explore this issue. Using medical history to collect sleep disorder information may have resulted in underreporting of sleep disorders. The lack of a gold standard for measuring treatment‐related changes in EDS in the PD population is another limitation, although not unique to this study. Finally, the MWT has not been validated in PD, hindering interpretation of the clinical relevance of these findings outside the context of clinically meaningful changes in other populations (eg, narcolepsy and OSA).

In conclusion, no new AEs or safety concerns were identified in participants with PD treated with solriamfetol, and there was no evidence that solriamfetol worsened PD motor symptoms. There were no statistical differences between solriamfetol and placebo on the ESS; however, a large placebo response was observed. Study design and conduct factors appear to have played confounding roles. As few studies have shown improvement in objective measures with other agents, the 5‐minute improvement in sleep latency on the MWT observed with 300 mg relative to placebo is notable. Larger, long‐term studies are needed to determine whether solriamfetol could represent an important treatment option for patients with PD.

## Financial Disclosures

P.K. Schweitzer has received consultancy fees from Jazz Pharmaceuticals and Apnimed. Her institution has received research funding from Apnimed, Avadel‐Flamel, Harmony Biosciences, Inspire Medical Systems, and Jazz Pharmaceuticals. R.A. Hauser has served as a consultant for AbbVie Inc., Acorda Therapeutics, Academy for Continued Healthcare Learning, Acadia Pharmaceuticals, Inc., Adamas Pharmaceuticals, AstraZeneca, ApoPharma, Back Bay Life Science, Biotie Therapies, Bracket, Cerecor, Inc., ClearView Healthcare Partners, ClinicalMind Medical and Therapeutic Communications, CNS Ratings, LLC, Cowen and Company, Cynapsus Therapeutics, DDB Health LLC, Decision Resources Group, Eli Lilly & Company, eResearch Technology, Inc., Expert Connect, Extera Partners, GE Healthcare, Health Advances, HealthLogix, Health and Wellness Partners, Huron Consulting Group, Impax Laboratories, Impel Neuropharma, Intec Pharma Ltd., Kashiv Pharma LLC, Kyowa Kirin Pharmaceutical Development, Ltd., LCN Consulting, LifeMax, Life Sciences, Lundbeck LLC, The Lockwood Group, MEDACorp, Medscape, Medtronic, The Michael J. Fox Foundation, Mitsubishi Tanabe Pharmaceuticals, Movement Disorder Society, National Institutes of Health (NIH), Neurocea LLC, Neurocrine Biosciences, Neuroderm, Neuropore Therapies, Orbes Medical Group, Outcomes Insights, Parkinson Study Group, Peerview Press, Pennside Partners, Pfizer, Inc., Pharma Two B, Ltd., Phase Five Communications, Prescott Medical Group, Prexton Therapeutics, Prilenia Development Ltd., Projects in Knowledge, Putnam Associates, Quintiles, RMEI Medical Education for Better Outcomes, SAI Med Partners LLC, Sarepta Therapeutics, Schlesinger Associates, Scion Neurostim, LLC, Seagrove Partners, LLC, Slingshot Insights, Sunovion Pharmaceuticals, Inc., Sun Pharma, Teva Pharmaceutical Industries, US WorldMeds, Vista Research, WebMD, and Windrose Consulting Group and research support from AbbVie Inc., Acorda Therapeutics, AstraZeneca, Axovant Sciences, Biogen Inc., Cavion, Enterin Inc., Impax Laboratories, LLC, Intec Pharma Ltd., Jazz Pharmaceuticals, NeuroDerm Ltd., Lundbeck, The Michael J. Fox Foundation for Parkinson's Research, F. Hoffman‐La Roche, Dart NeuroScience LLC, Prexton Therapeutics, Revance Therapeutics Inc., Sunovion Pharmaceuticals, and grant support from the Parkinson's Foundation. A.W. Amara has received grant funding from NIH, serves as site investigator for studies sponsored by the The Michael J. Fox Foundation for Parkinson's Research, Biogen Idec, Hoffman‐La Roche, Eli Lilly, Axovant Sciences, Ltd., and AbbVie Laboratories, and serves as a consultant for Grey Matter Technologies. A. Videnovic has received research funding from NIH, has served on a Data Safety Monitoring Board for Acorda Therapeutics and Wilson's Therapeutics. C. Comella serves on the editorial board of *Clinical Neuropharmacology and Sleep Medicine*, receives research support from the NIH R01NS074343, U54NS065701, Dystonia Medical Research Foundation, Merz Pharmaceutical, Revance Therapeutic, Retrophin, and Acorda Therapeutic; receives compensation/honoraria for services as a consultant or an advisory committee member from Acorda Therapeutics, Aeon Pharmaceutical, Allergan, Inc., Lundbeck Ltd., Ipsen, Merz Pharmaceuticals, Acadia Pharmaceuticals, Neurocrine Biosciences Inc., and Revance Therapeutic; receives royalties from Cambridge and Wolters Kluwer; and receives research support from the Parkinson's Disease Foundation. H. Emsellem has received research funding from Vanda Pharmaceuticals, Eisai, Flamel, Balance Therapeutics, Harmony Bioscience, Idorsia, Imbrium Therapeutics, Takeda Pharmaceuticals, Expansion Therapeutics, and Merck & Co., Inc.; and was a board member of the National Sleep Foundation through June 30, 2019.

## Author Roles

This clinical research was funded by Jazz Pharmaceuticals (the sponsor), which also took a leadership role in designing the study. All of the authors, including authors from Jazz Pharmaceuticals, assisted in the collection, analysis, and interpretation of data, in the writing of the report, and in the decision to submit the paper for publication.

## Supporting information


**Appendix S1**: Supplementary Information.

## Data Availability

All relevant data are provided with the manuscript and supporting files.
