# Supplementary material for: Solriamfetol for Excessive Daytime Sleepiness in Parkinson's Disease: Phase 2 Proof‐of‐Concept Trial
Source: Mov Disord. 2021 Jun 30;36(10):2408–12. doi: 10.1002/mds.28702 (PMC8596433; doi:10.1002/mds.28702)
Supplement: Supplementary file 1 — Appendix S1: Supplementary Information. [file MDS-36-2408-s001.docx]

**SUPPLEMENTARY MATERIALS**

**Methods**

*Study Design*

The study was approved by institutional review boards or ethics committees at each site and performed in accordance with the Declaration of Helsinki; all participants provided written informed consent. Randomization was performed via an automated interactive voice/web response system with the master randomization code sequestered by the sponsor.

*Patients and Methods*

Participants were adults aged 35 to 80 years with PD (United Kingdom Parkinson’s Disease Society Brain Bank criteria), and EDS (Epworth Sleepiness Scale [ESS] score >11). Additional inclusion criteria included Hoehn and Yahr stage 1–3, and a stable PD treatment regimen (ie, medications for treatment of motor and nonmotor symptoms) for 4 weeks prior to screening. Participants were excluded if they had a diagnosis of another degenerative Parkinsonian syndrome; history of deep brain stimulation; usual overnight time in bed <6 hours; occupation requiring nighttime or variable shift work; any other clinically relevant disorder associated with EDS; severe cognitive impairment (Scales for Outcomes in Parkinson’s Disease–Cognition [SCOPA-COG] score ≤24); impulse control disorder (Questionnaire for Impulsive-Compulsive Disorders in Parkinson’s Disease-Rating Scale total score >30 or score >10 in individual components of gambling, buying, sex, or eating); or use of medications that could affect evaluation of EDS (eg, stimulants, sodium oxybate, diphenhydramine) within 14 days of baseline or during the study. Participants also were excluded if they had evidence of untreated or inadequately treated moderate or severe OSA as determined by a documented history or a positive score in both categories 1 and 3 on the Berlin Sleep Apnea Questionnaire at screening. However, participants with a positive Berlin Sleep Apnea Questionnaire score were eligible if they underwent additional testing for OSA and did not meet the following criteria: apnea index >10 events/hour (assessed by in-laboratory polysomnography or home sleep test), or oxygen desaturation index >20 events/hour with ≥3% drop in oxygen saturation (assessed by overnight pulse oximetry). These assessments were performed either during screening, at the baseline visit, or by historical evaluation performed within 12 months of screening and accompanied by ≤5% increase in weight. Trazodone, benzodiazepines, nonbenzodiazepines, suvorexant, and melatonin receptor agonists were permitted for treatment of insomnia if used on a stable regimen 14 days prior to baseline and throughout the study.

*Outcome Measures*

For the ESS, participants were instructed to assess their sleepiness during the previous week (1-week recall period). PK samples were collected predose, and at 1, 2, 3, 4, 5, 6, and (if feasible) 7 to 8 hours after dosing at the end of each 1-week treatment period in a subset of participants who enrolled prior to implementation of Protocol Amendment 2 (which removed PK assessments). The MWT was conducted in a subset of participants (Protocol Amendment 2 made the MWT optional). The MWT, a laboratory-based measure, typically consists of four 40-minute trials (separated by 2-hour intervals),^1^ during which participants try to remain awake in a soporific environment.^1^ It was chosen instead of the Multiple Sleep Latency Test (MSLT), which assesses ability to fall asleep in a sleep-inducing environment, because the ability to remain awake is more relevant in determining response to a wake-promoting agent. Additionally, the MWT is less subject to a “floor effect” in participants with severe sleepiness and has greater sensitivity to medication effects than the MSLT.^1,2^ To minimize participants’ burden, a 3-trial MWT was used (fourth trial optional), and in-laboratory nocturnal polysomnography before the MWT was not required.^1,2^ While previous research in healthy volunteers suggested a 3-trial MSLT provides similar test-retest reliability to a 4-trial MSLT,^3^ this 3-trial MWT protocol has not been standardized.

*Statistical Analysis*

Safety data were summarized descriptively by treatment. Differences from placebo in change from baseline on ESS, MWT, MDS-UPDRS Parts III and IV, FSS, Apathy Scale, and SCOPA-COG were analyzed using mixed-effects models with fixed effects for treatment group, sequence, treatment-by-sequence interaction, and baseline value of the efficacy endpoint and a random effect for participant. PGI-C and CGI-C were analyzed using χ^2^ tests; ratings were dichotomized into 2 categories: improved (*very much improved*, *much improved*, and *minimally improved* categories) and not improved (*no change*, *minimally worse*, *much worse*, and *very much worse* categories). No adjustments for multiplicity were performed; therefore, *P* values are nominal. Plasma PK parameters were calculated for solriamfetol concentrations by noncompartmental methods and were summarized by dose level using descriptive statistics.

Baseline characteristics and safety data were analyzed for the safety population (participants who took ≥1 dose of study drug). Efficacy data were analyzed for the modified intent-to-treat (mITT) population (randomized participants who received ≥1 dose of study drug, and had a baseline and ≥1 postbaseline efficacy assessment). Efficacy data were also analyzed for the per protocol (PP) population, which consisted of the mITT population except those with major protocol deviations that could impact efficacy assessments. Efficacy data are presented by dose level and a single pooled placebo group (combination of placebo groups from each treatment sequence). To account for potential carryover effects on ESS from the 300-mg to placebo period in sequence B, given the 1-week recall period and the lack of washout between treatment periods, a prespecified sensitivity analysis for the ESS excluded sequence B placebo data. A similar post hoc analysis was performed for PGI-C. Post hoc exploratory analyses of ESS scores, MWT mean sleep latency, and treatment-emergent AEs (TEAEs) were conducted for subgroups based on dopamine agonist use. An additional post hoc exploratory analysis for the PGI-C was conducted using a higher threshold for improvement (*very much* and *much improved*).

**References**

1. Littner MR, Kushida C, Wise M, et al. Practice parameters for clinical use of the Multiple Sleep Latency Test and the Maintenance of Wakefulness Test. Sleep 2005;28(1):113-121.

2. Randomized trial of modafinil for the treatment of pathological somnolence in narcolepsy. US Modafinil in Narcolepsy Multicenter Study Group. Ann Neurol 1998;43(1):88-97.

3. Zwyghuizen-Doorenbos A, Roehrs T, Schaefer M, Roth T. Test-retest reliability of the MSLT. Sleep 1988;11(6):562-565.

**Supplemental Figures**

**A.**


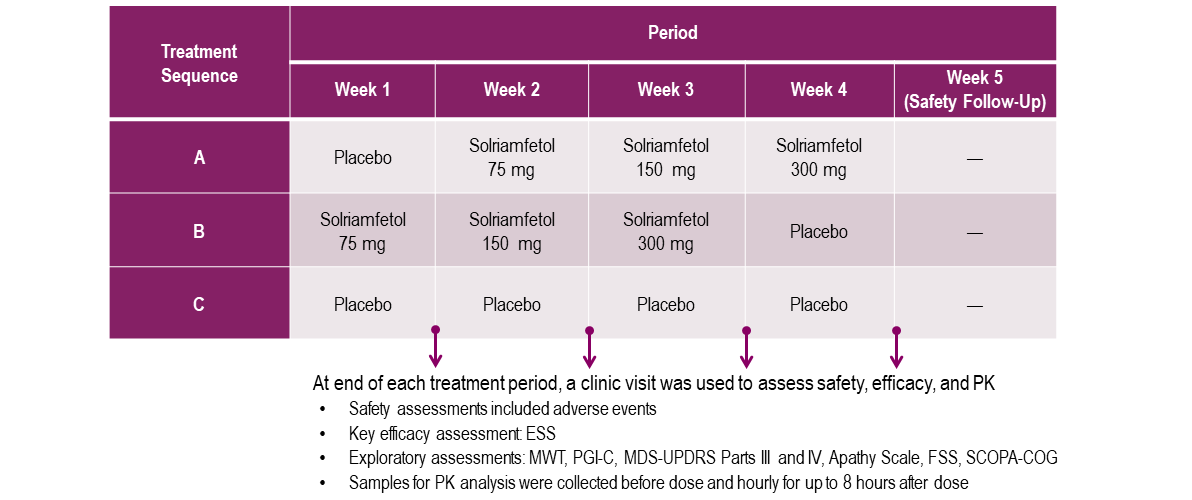


**B.**
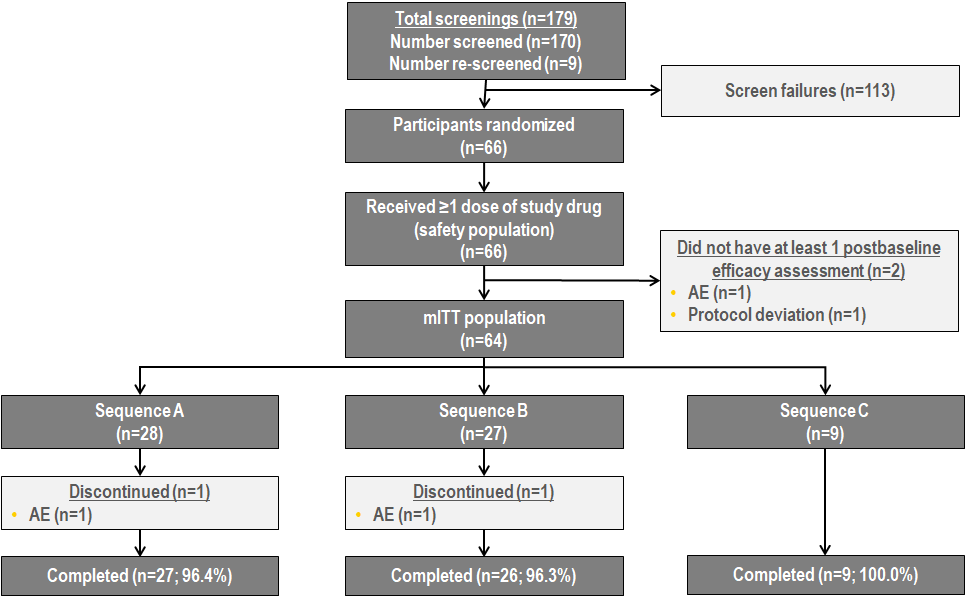

**Figure S1A-B**. (A) Study design. Participants were randomized (3:3:1) to 3 sequence groups (A, B, C) for 4 weeks of treatment; there was no washout between 1-week treatment periods.
(B) Participant disposition.

AE, adverse event; ESS, Epworth Sleepiness Scale; FSS, Fatigue Severity Scale; MDS-UPDRS, Movement Disorders Society–Unified Parkinson’s Disease Rating Scale; mITT, modified intent-to-treat; MWT, Maintenance of Wakefulness Test; PGI-C, Patient Global Impression of Change; PK, pharmacokinetics; SCOPA-COG, Scales for Outcomes in Parkinson’s Disease–Cognition.

**A.**


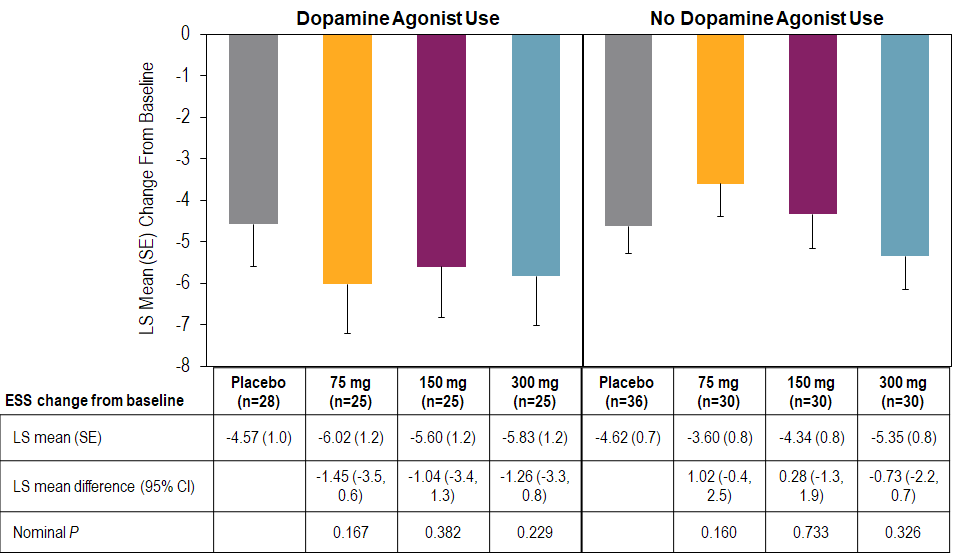


**B.**


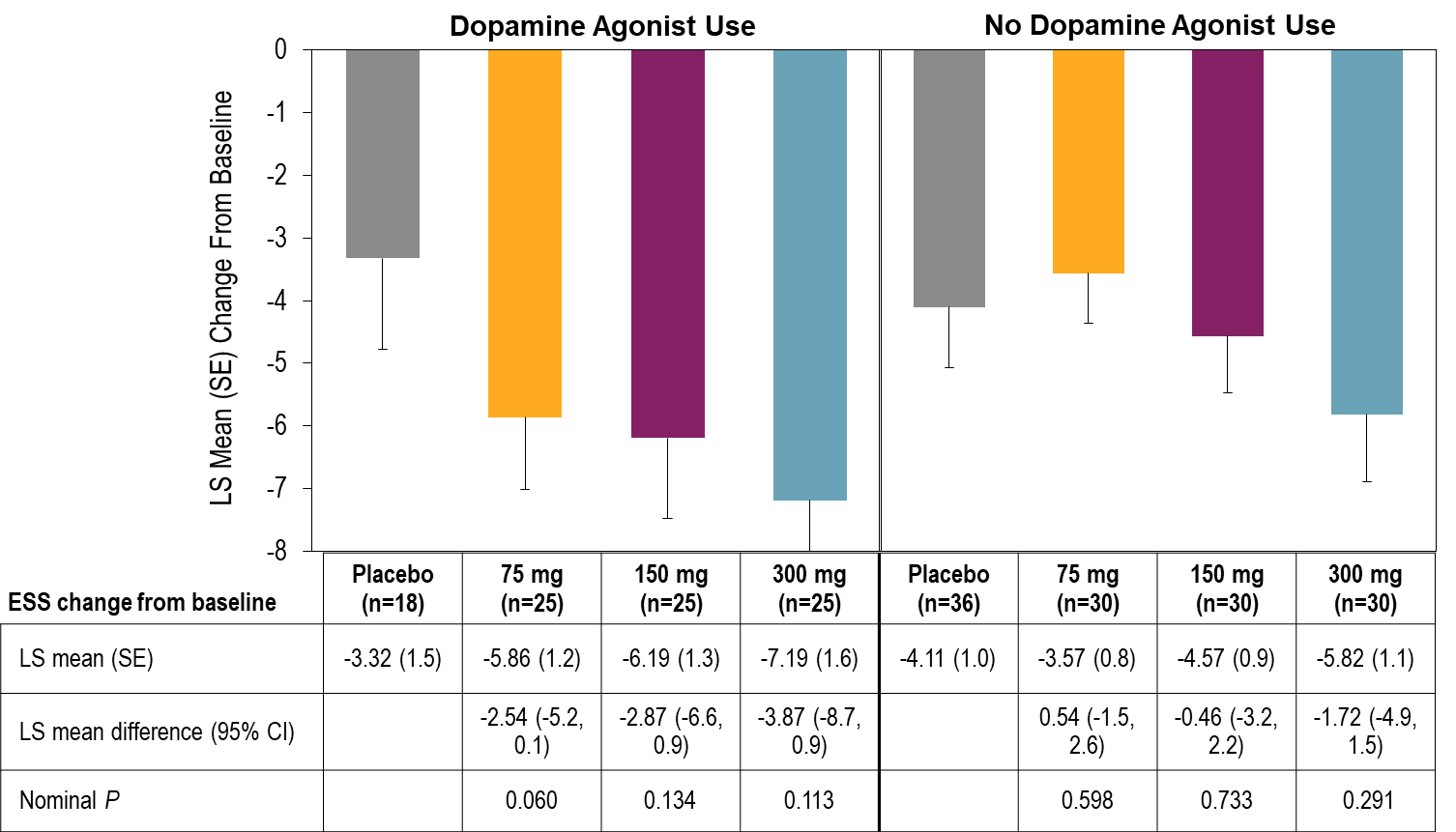


**Figure S2A-B.** Change from baseline on the secondary efficacy endpoint, ESS scores (modified intent-to-treat population). (A) Post hoc analysis by dopamine agonist use (all participants). (B) Post hoc analysis by dopamine agonist use (excluding sequence B placebo).

CI, confidence interval; ESS, Epworth Sleepiness Scale; LS, least squares; SE, standard error.

^
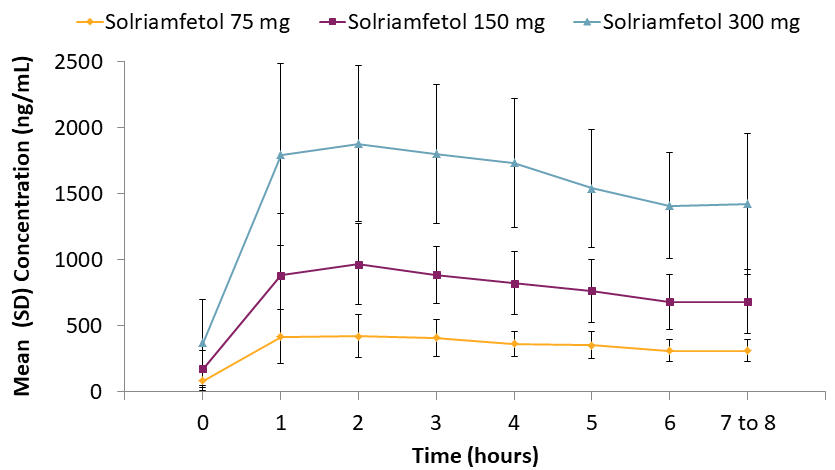
^

**Figure S3**. Mean (SD) solriamfetol plasma concentrations over time (n=19).

SD, standard deviation.

**Table S1**. Baseline demographic and clinical characteristics (safety population)

| **Characteristic** | **Treatment Sequence^a^** | | | **Overall**  **(N=66)** |
| --- | --- | --- | --- | --- |
|  | **A**  **(n=28)** | **B**  **(n=28)** | **C**  **(n=10)** |  |
| Age (y), mean (SD) | 65.9 (7.6) | 62.9 (8.8) | 65.4 (9.8) | 64.6 (8.5) |
| Sex (male), n (%) | 18 (64.3) | 22 (78.6) | 5 (50.0) | 45 (68.2) |
| BMI (kg/m^2^), mean (SD) | 29.1 (4.5) | 28.1 (4.4) | 30.3 (5.4) | 28.9 (4.6) |
| Years since PD diagnosis, mean (SD) | 7.3 (4.5) | 6.5 (4.8) | 4.3 (2.6) | 6.5 (4.5) |
| Hoehn & Yahr stage, n (%) |  |  |  |  |
| 1 | 1 ( 3.6) | 2 ( 7.1) | 0 | 3 (4.5) |
| 2 | 20 (71.4) | 21 (75.0) | 9 (90.0) | 50 (75.8) |
| 3 | 7 (25.0) | 5 (17.9) | 1 (10.0) | 13 (19.7) |
| ESS score, mean (SD) | 16.1 (2.9) | 16.5 (2.8) | 14.8 (3.2) | 16.1 (2.9) |
| MWT sleep latency | n=24 | n=24 | n=4 | n=52 |
| Mean (SD) (min) | 14.3 (10.7) | 15.6 (11.1) | 16.3 (13.3) | 15.1 (10.9) |
| SCOPA-COG total score, mean (SD) | 31.8 (4.3) | 31.2 (4.0) | 29.3 (4.9) | 31.2 (4.3) |
| MDS-UPDRS score, mean (SD) |  |  |  |  |
| Part III total score^b^ | 26.6 (11.6) | 31.4 (10.1) | 28.5 (14.0) | 28.9 (11.4) |
| Part IV total score^c^ | 3.6 (3.2) | 3.0 (2.7) | 4.3 (4.9) | 3.4 (3.3) |
| Apathy Scale total score, mean (SD) | 10.3 (6.4) | 11.3 (6.4) | 10.4 (5.5) | 10.7 (6.2) |
| FSS total score | n=27 | n=28 | n=9 | n=64 |
| Mean (SD) | 4.3 (1.5) | 4.2 (1.4) | 3.9 (1.6) | 4.2 (1.5) |
| Levodopa/dopamine agonist use, n (%) |  |  |  |  |
| Levodopa only | 13 (46.4) | 18 (64.3) | 7 (70.0) | 38 (57.6) |
| Levodopa + dopamine agonist | 14 (50.0) | 8 (28.6) | 3 (30.0) | 25 (37.9) |
| Dopamine agonist only | 1 (3.6) | 2 (7.1) | 0 | 3 (4.5) |
| Total LED (mg),^d^ mean (SD) | 613.1 (278.3) | 669.6 (333.7) | 520.4 (256.2) | 623.0 (300.2) |
| MAO inhibitor use, n (%) | 11 (39.3) | 9 (32.1) | 0 | 20 (30.3) |

^a^Sequence A: placebo, 75, 150, 300 mg; Sequence B: 75, 150, 300 mg, placebo; Sequence C: 4 weeks placebo. ^b^Sum of 33 items rated on 0-4 scale (0=normal; 4=severe).^c^Sum of 6 items rated on 0-4 scale (0=normal; 4=severe). ^d^The sum of the total daily dose of each PD motor symptoms-related medication, converted into an LED expressed in mg.

BMI, body mass index; ESS, Epworth Sleepiness Scale; FSS, Fatigue Severity Scale; LED, levodopa equivalent dose; MDS-UPDRS, Movement Disorders Society–Unified Parkinson’s Disease Rating Scale; mg, milligrams; MWT, Maintenance of Wakefulness Test; PD, Parkinson’s disease; SCOPA-COG, Scales for Outcomes in Parkinson’s Disease–Cognition; SD, standard deviation.

**Table S2.** Treatment-emergent adverse events by dopamine agonist use at baseline (safety population)

| **Participants With ≥1 TEAE, n (%)** | **Dopamine Agonists^a^** | | | | | **No Dopamine Agonists^a^** | | | | |
| --- | --- | --- | --- | --- | --- | --- | --- | --- | --- | --- |
|  | **Placebo^b^**  **(n=28)** | **Solriamfetol** | | | | **Placebo^b^**  **(n=36)** | **Solriamfetol** | | | |
|  |  | **75 mg**  **(n=25)** | **150 mg**  **(n=25)** | **300 mg**  **(n=25)** | **Combined^c^**  **(n=25)** |  | **75 mg**  **(n=31)** | **150 mg**  **(n=30)** | **300 mg**  **(n=29)** | **Combined^c^**  **(n=31)** |
| Any TEAE | 4 (14.3) | 7 (28.0) | 7 (28.0) | 8 (32.0) | 12 (48.0) | 12 (33.3) | 11 (35.5) | 13 (43.3) | 7 (24.1) | 21 (67.7) |
| Serious TEAE | 0 | 0 | 0 | 0 | 0 | 0 | 0 | 0 | 1 (3.4) | 1 (3.2) |
| Discontinuation due to TEAE^d^ | 0 | 0 | 0 | 0 | 0 | 0 | 1 (3.2) | 2 (6.7) | 0 | 3 (9.7) |
| Common TEAEs (≥5%) | | | | | | | | | | |
| Dry mouth | 1 (3.6) | 2 (8.0) | 2 (8.0) | 0 | 4 (16.0) | 1 (2.8) | 0 | 0 | 0 | 0 |
| Dyspepsia | 0 | 2 (8.0) | 0 | 0 | 2 (8.0) | 0 | 0 | 1 (3.3) | 0 | 1 (3.2) |
| Falls | 0 | 1 (4.0) | 0 | 1 (4.0) | 2 (8.0) | 0 | 0 | 0 | 0 | 0 |
| Dizziness | 0 | 2 (8.0) | 0 | 0 | 2 (8.0) | 0 | 1 (3.2) | 0 | 1 (3.4) | 2 (6.5) |
| Headache | 0 | 1 (4.0) | 2 (8.0) | 1 (4.0) | 2 (8.0) | 0 | 0 | 1 (3.3) | 1 (3.4) | 2 (6.5) |
| Nausea | 0 | 1 (4.0) | 0 | 0 | 1 (4.0) | 0 | 1 (3.2) | 2 (6.7) | 3 (10.3) | 5 (16.1) |
| Constipation | 0 | 0 | 0 | 0 | 0 | 0 | 2 (6.5) | 1 (3.3) | 0 | 3 (9.7) |
| Blood pressure increased | 0 | 0 | 0 | 0 | 0 | 1 (2.8) | 1 (3.2) | 0 | 1 (3.4) | 2 (6.5) |
| Anxiety | 0 | 1 (4.0) | 0 | 0 | 1 (4.0) | 2 (5.6) | 0 | 2 (6.7) | 0 | 2 (6.5) |

Note: A total of 28 participants were taking a dopamine agonist at baseline (sequence A, n=15; sequence B, n=10; sequence C, n=3). ^a^At baseline. ^b^Pooled across all sequences. ^c^Pooled across all solriamfetol doses (sequences A and B only). ^d^TEAE leading to early discontinuation of study drug and/or to study withdrawal.
TEAE, treatment-emergent adverse event.

**Table S3.** Changes from baseline in orthostatic blood pressure (safety population)

| Change from seated to standing, 3 min after standing | **Solriamfetol** | | | **Placebo** | | | |
| --- | --- | --- | --- | --- | --- | --- | --- |
|  | **75 mg**  **(N=56)** | **150 mg**  **(N=55)** | **300 mg**  **(N=54)** | **Sequence A (N=28)** | **Sequence B**  **(N=26)** | **Sequence C (N=10)** | **Pooled**  **(N=64)** |
| **Systolic Blood Pressure (mmHg)** | | | | | | | |
| Change from baseline (0-hour) to end of treatment (pre-dose) | n=51 | n=51 | n=50 | n=26 | n=25 | n=9 | n=60 |
| Mean (SD) | 0.6 (11.1) | -1.4 (10.4) | -2.3 (10.5) | 1.1 (9.6) | -4.4 (11.2) | -0.8 (5.6) | -1.5 (10.0) |
| Median (min, max) | 1.5 (-26.0, 25.3) | -1.0 (-25.0, 24.6) | -3.0 (-22.0, 24.6) | -0.3 (-19.0, 18.5) | -6.0 (-22.0, 30.3) | -0.5 (-8.4, 10.0) | -2.0 (-22.0, 30.3) |
| Change from baseline (2-hour) to end of treatment (1-hour post-dose) | n=50 | n=52 | n=49 | n=27 | n=24 | n=9 | n=60 |
| Mean (SD) | 1.8 (9.0) | 0.7 (9.8) | 0.1 (10.1) | 1.7 (7.9) | 0.6 (7.0) | -2.9 (6.2) | 0.6 (7.4) |
| Median (min, max) | 1.0 (-19.0, 31.3) | 0.5 (-30.7, 18.5) | -1.0 (-20.5, 37.5) | 1.0 (-16.7, 18.5) | 0.8 (-11.0, 17.5) | -3.0 (-12.8, 7.5) | -0.4 (-16.7, 18.5) |
| Change from baseline (3-hour) to end of treatment (2-hour post-dose) | n=52 | n=52 | n=50 | n=27 | n=25 | n=8 | n=60 |
| Mean (SD) | -2.3 (10.1) | -4.8 (12.7) | -5.8 (13.2) | -4.1 (13.0) | -4.7 (13.0) | 2.2 (8.3) | -3.5 (12.5) |
| Median (min, max) | -1.7 (-34.2, 23.0) | -3.3 (-38.4, 23.0) | -4.2 (-47.2, 21.0) | -2.5 (-41.4, 16.5) | -6.7 (-25.4, 20.0) | 1.3 (-7.1, 21.2) | -2.0 (-41.4, 21.2) |
| Change from baseline (5-hour) to end of treatment (4-hour post-dose) | n=44 | n=44 | n=43 | n=23 | n=21 | n=6 | n=50 |
| Mean (SD) | 0.4 (10.9) | -1.4 (10.6) | -1.2 (13.2) | -0.8 (9.8) | -2.3 (11.0) | 2.8 (12.9) | -1.0 (10.5) |
| Median (min, max) | 1.8 (-19.7, 23.5) | -1.6 (-28.0, 16.0) | 0.5 (-31.8, 25.0) | -3.0 (-14.0, 24.5) | 1.0 (-27.0, 16.5) | -0.9 (-7.7, 28.4) | -1.2 (-27.0, 28.4) |
| **Diastolic Blood Pressure (mmHg)** | | | | | | | |
| Change from baseline (0-hour) to end of treatment (pre-dose) | n=51 | n=51 | n=50 | n=26 | n=25 | n=9 | n=60 |
| Mean (SD) | 0.4 (7.6) | 0.3 (9.3) | 0.9 (7.8) | 0.6 (5.5) | 0.0 (6.8) | -1.9 (3.8) | 0.0 (5.9) |
| Median (min, max) | 0.3 (-14.2, 23.8) | 1.4 (-24.0, 20.3) | 0.0 (-14.2, 19.5) | -0.4 (-8.5, 13.5) | 0.5 (-16.0, 14.7) | -1.1 (-8.5, 5.8) | -0.8 (-16.0, 14.7) |
| Change from baseline (2-hour) to end of treatment (1-hour post-dose) | n=50 | n=52 | n=49 | n=27 | n=24 | n=9 | n=60 |
| Mean (SD) | 0.8 (6.1) | -0.4 (6.4) | 0.9 (6.6) | 0.6 (7.4) | 1.4 (5.9) | -0.4 (5.9) | 0.8 (6.5) |
| Median (min, max) | 1.3 (-14.0, 15.5) | -0.3 (-17.5, 17.5) | 0.0 (-16.7, 17.5) | 0.0 (-13.5, 27.5) | 1.0 (-9.0, 14.5) | -0.7  (-9.2, 6.8) | 0.0 (-13.5, 27.5) |
| Change from baseline (3-hour) to end of treatment (2-hour post-dose) | n=52 | n=52 | n=50 | n=27 | n=25 | n=8 | n=60 |
| Mean (SD) | -0.8 (8.8) | -1.3 (9.4) | -1.8 (10.1) | 0.8 (9.0) | -0.4 (8.1) | 0.1 (7.4) | 0.2 (8.3) |
| Median (min, max) | -0.5 (-17.5, 31.3) | -0.6 (-30.4, 32.0) | -1.3 (-23.5, 30.8) | 0.0 (-16.2, 30.6) | 0.0 (-15.0, 18.0) | 1.5 (-12.8, 11.7) | 0.0 (-16.2, 30.6) |
| Change from baseline (5-hour) to end of treatment (4-hour post-dose) | n=44 | n=44 | n=43 | n=23 | n=21 | n=6 | n=50 |
| Mean (SD) | 0.8 (7.9) | 1.0 (6.7) | 0.0 (8.7) | 1.7 (6.6) | -1.1 (10.9) | -2.3 (3.2) | 0.1 (8.5) |
| Median (min, max) | 2.0 (-13.0, 26.6) | 0.2 (-11.0, 21.8) | 0.0 (-18.5, 23.5) | 1.7 (-14.0, 13.3) | -2.0 (-27.5, 18.0) | -2.3 (-5.6, 3.1) | 0.2 (-27.5, 18.0) |

SD, standard deviation.

**Table S4**. Exploratory outcomes (mITT population)

| **Change From Baseline Scores** | **Placebo** | **Solriamfetol** | | |
| --- | --- | --- | --- | --- |
|  |  | **75 mg** | **150 mg** | **300 mg** |
| **MWT mean sleep latency, change from baseline, min^a^** |  |  |  |  |
| All participants | n=45 | n=42 | n=43 | n=41 |
| LS mean (SE) | 1.77 (1.9) | 0.43 (2.1) | 2.67 (2.2) | 6.81 (2.1) |
| LS mean difference  (95% CI) |  | –1.34  (–5.08, 2.40) | 0.91  (–3.38, 5.19) | 5.05  (1.24, 8.57) |
| Nominal *P* |  | 0.480 | 0.677 | 0.0098 |
| Participants receiving dopamine agonists at baseline | n=20 | n=19 | n=19 | n=19 |
| LS mean (SE) | 2.21 (3.1) | –2.09 (3.5) | 0.16 (3.6) | 4.83 (3.5) |
| LS mean difference  (95% CI) |  | –4.30  (–10.31, 1.71) | –2.05  (–8.92, 4.82) | 2.62 (–3.35, 8.59) |
| Nominal *P* |  | 0.157 | 0.552 | 0.383 |
| Participants not receiving dopamine agonists at baseline | n=33 | n=28 | n=28 | n=28 |
| LS mean (SE) | 1.02 (2.2) | 1.74 (2.6) | 3.38 (2.7) | 7.54 (2.6) |
| LS mean difference  (95% CI) |  | 0.71  (–4.27, 5.70) | 2.36  (–3.38, 8.10) | 6.52  (1.31, 11.72) |
| Nominal *P* |  | 0.775 | 0.415 | 0.015 |
| **PGI-C** | n=64 | n=55 | n=55 | n=55 |
| Prespecified definition of improvement |  |  |  |  |
| Improved (*minimally, much,* or *very much improved*), n (%) | 39 (60.9) | 29 (52.7) | 33 (60.0) | 36 (65.5) |
| Not improved, n (%) | 24 (37.5) | 26 (47.3) | 20 (36.4) | 17 (30.9) |
| Nominal *P* |  | 0.3142 | 0.9683 | 0.4993 |
| Post hoc definition of improvement |  |  |  |  |
| Improved (*much* or *very much improved*), n (%) | 12 (18.8) | 11 (20.0) | 18 (32.7) | 18 (32.7) |
| Not improved, n (%) | 51 (79.7) | 44 (80.0) | 35 (63.6) | 35 (63.6) |
| Nominal *P* |  | 0.8963 | 0.0676 | 0.0676 |
| Sensitivity analysis excluding sequence B placebo | n=37 | n=55 | n=55 | n=55 |
| Improved (*minimally, much,* or *very much improved*), n (%) | 20 (54.1) | 29 (52.7) | 33 (60.0) | 36 (65.5) |
| Not improved, n (%) | 17 (45.9) | 26 (47.3) | 20 (36.4) | 17 (30.9) |
| Nominal *P* |  | 0.9005 | 0.4361 | 0.1817 |
| **CGI-C** | n=64 | n=55 | n=55 | n=55 |
| Improved (*minimally, much,* or *very much improved*), n (%) | 39 (60.9) | 31 (56.4) | 35 (63.6) | 37 (67.3) |
| Not improved, n (%) | 24 (37.5) | 23 (41.8) | 19 (34.5) | 16 (29.1) |
| Nominal *P* |  | 0.6208 | 0.7448 | 0.3721 |
| **MDS-UPDRS Part III, change from baseline** | n=62 | n=55 | n=54 | n=52 |
| LS mean (SE) | –1.97 (1.03) | –0.92 (1.28) | –0.49 (1.35) | –2.17 (1.30) |
| LS mean difference  (95% CI) |  | 1.06 (–1.56, 3.68) | 1.49 (–1.48, 4.45) | –0.20 (–2.84, 2.44) |
| Nominal *P* |  | 0.4268 | 0.3242 | 0.8807 |
| **MDS-UPDRS Part IV, change from baseline** | n=61 | n=54 | n=53 | n=51 |
| LS mean (SE) | 0.25 (0.31) | –0.71 (0.37) | –0.97 (0.39) | 0.27 (0.38) |
| LS mean difference  (95% CI) |  | –0.96 (–1.69, –0.23) | –1.23 (–2.05, –0.40) | 0.02 (–0.72, 0.76) |
| Nominal *P* |  | 0.0106 | 0.0040 | 0.9589 |
| **Apathy Scale, change from baseline** | n=63 | n=55 | n=54 | n=53 |
| LS mean (SE) | –0.19 (0.52) | –1.47 (0.62) | –0.31 (0.64) | –0.05 (0.62) |
| LS mean difference  (95% CI) |  | –1.28 (–2.45, –0.12) | –0.12 (–1.44, 1.19) | 0.14 (–1.03, 1.31) |
| Nominal *P* |  | 0.0305 | 0.8528 | 0.8129 |
| **FSS, change from baseline** | n=61 | n=54 | n=53 | n=52 |
| LS mean (SE) | –0.30 (0.51) | –0.28 (0.81) | –0.53 (0.19) | –0.25 (0.18) |
| LS mean difference  (95% CI) |  | 0.02 (–0.34, 0.38) | –0.23 (–0.64, 0.18) | 0.05 (–0.32, 0.42) |
| Nominal *P* |  | 0.9172 | 0.2698 | 0.7825 |
| **SCOPA-COG, change from baseline** | n=63 | n=55 | n=54 | n=53 |
| LS mean (SE) | 1.93 (0.45) | 2.45 (0.52) | 2.07 (0.54) | 2.22 (0.52) |
| LS mean difference  (95% CI) |  | 0.52 (–0.36, 1.39) | 0.14 (–0.85, 1.13) | 0.29 (–0.60, 1.17) |
| Nominal *P* |  | 0.2459 | 0.7862 | 0.5249 |

^a^The majority of participants (n=42) completed a 3-trial MWT, whereas the rest (n=11) completed a 4-trial MWT.

CGI-C, Clinician Global Impression of Change; CI, confidence interval; FSS, Fatigue Severity Scale; LS, least squares; MDS-UPDRS, Movement Disorder Society–Unified Parkinson’s Disease Rating Scale; mITT, modified intent-to-treat; MWT, Maintenance of Wakefulness Test; PGI-C, Patient Global Impression of Change; SCOPA-COG, Scales for Outcomes in Parkinson’s Disease–Cognition; SE, standard error.

**Table S5**. Per protocol analysis: change from baseline on the ESS and MWT (per protocol population)^a^

|  |  | **Solriamfetol** | | |
| --- | --- | --- | --- | --- |
| **Parameter (units)** | **Placebo^b^ (n=53)** | **75 mg^b^ (n=45)** | **150 mg^b^ (n=45)** | **300 mg^b^ (n=45)** |
| **ESS change from baseline** | | | | |
| n | 52 | 45 | 44 | 43 |
| LS mean (SE) | -4.87 (0.64) | -4.95 (0.75) | -5.22 (0.78) | -6.24 (0.75) |
| LS mean difference  (95% CI) |  | -0.08  (-1.44, 1.27) | -0.35  (-1.88, 1.18) | -1.38  (-2.75, -0.01) |
| Nominal *P* |  | 0.9033 | 0.6515 | 0.0483 |
| **MWT mean sleep latency change from baseline** | | | | |
| n | 39 | 37 | 37 | 35 |
| LS mean (SE) | 1.66 (1.99) | 0.80 (2.22) | 3.15 (2.29) | 7.58 (2.24) |
| LS mean difference  (95% CI) |  | -0.86  (-4.49, 2.78) | 1.50  (-2.68, 5.67) | 5.92  (2.16, 9.68) |
| Nominal *P* |  | 0.6414 | 0.4792 | 0.0023 |

^a^From the mITT population, a total of 11 participants (sequence A, n=4; sequence B, n=6; sequence C, n=1) were excluded from the per protocol population; the most common reason for deviation was dosing error; participants received either an incorrect dose or no dose on one or more clinic visit days when efficacy assessments were taken (n=8; sequence A, n=3; sequence B, n=4; sequence C, n=1). ^b^Pooled across all sequences.

CI, confidence interval; ESS, Epworth Sleepiness Scale; LS, least squares; mITT, modified intent-to-treat; MWT, Maintenance of Wakefulness Test; SE, standard error.

**Table S6.** Solriamfetol plasma PK parameters (PK population)

|  | **Solriamfetol** | | |
| --- | --- | --- | --- |
| **Parameter (units)** | **75 mg (n=16)** | **150 mg (n=16)** | **300 mg (n=18)** |
| C_max_ (ng/mL), mean (SD) | 495.8 (159.1) | 1063 (315.7) | 2018 (572.5) |
| T_max_ (h), median (range) | 1.1 (0.9–7.0) | 1.9 (0.9–3.1) | 2.0 (0.9–3.0) |
| t_1/2_ (h), mean (SD) | 8.0 (2.5) | 8.5 (2.9) | 9.5 (5.9) |
| AUC_0-t_ (ng*h/mL), mean (SD) | 2481 (822.8) | 5426 (1837) | 10,920 (3,641) |
| CL/F (L/h), mean (SD) | 16.3 (5.8) | 15.5 (5.0) | 15.4 (5.9) |
| V_d_/F (L), mean (SD) | 174.6 (41.3) | 178.0 (45.6) | 176.8 (40.5) |

AUC_0-t_, area under concentration-time curve from zero to time t; CL/F, apparent plasma clearance after oral administration; C_max_, observed maximum plasma concentration; PK, pharmacokinetics; SD, standard deviation; t_1/2_, terminal half-life; T_max_, time to reach maximum plasma concentration; V_d_/F, apparent volume of distribution.
